# Having siblings promotes a more healthy weight status—Whereas only children are at greater risk for higher BMI in later childhood

**DOI:** 10.1371/journal.pone.0271676

**Published:** 2022-07-19

**Authors:** Claudia Bohn, Mandy Vogel, Tanja Poulain, Andreas Hiemisch, Wieland Kiess, Antje Körner

**Affiliations:** 1 Medical Faculty, LIFE Child (Leipzig Research Centre for Civilization Diseases), Leipzig University, Leipzig, Germany; 2 Department of Women and Child Health, Hospital for Children and Adolescents and Centre for Paediatric Research (CPL), Medical Faculty, Leipzig University, Leipzig, Germany; Bavarian Health and Food Safety Authority: Bayerisches Landesamt fur Gesundheit und Lebensmittelsicherheit, GERMANY

## Abstract

**Background:**

Birth order and having at least one sibling are known to be associated with an increased risk for development of overweight. However, there are no studies assessing pre- and postnatal factors for developing overweight within families. Therefore, the present study aimed to analyse the association of the mother’s weight gain during pregnancy, prepregnancy BMI, mother’s age at birth, breastfeeding, age gap between siblings, and physical activity together with sibling-related characteristics on the development of overweight in children and adolescents.

**Methods:**

Data were obtained from the longitudinal LIFE Child cohort. The study sample included n = 1932 children, stratified into first-born (n = 578), second-born (n = 608), third-or-later-born single-born siblings (n = 162), only children (n = 526), and twin children (n = 58). Children with chronic or syndromic diseases, born prematurely or from mothers with gestational diabetes were excluded. Data were adjusted for multiple children per family using mixed models. Pregnancy weight gain, prepregnancy BMI and mother’s age were considered prenatal co-variates. Postnatal factors included the duration of breastfeeding and the children’s physical activity level.

**Results:**

Particularly until the onset of puberty, the BMI-SDS differed between single-born siblings, only children and twins, and increased with birth order. Compared to children with siblings, only children exhibited a strong increase in BMI-SDS starting at age nine. A higher age gap between siblings was associated with a higher BMI-SDS in second- and third-or-later-born children. Single-born siblings had the highest rate and duration of breastfeeding. Physical activity was highest in twins and third-or-later-born children and lowest in only children. In a multivariate model, being an only child showed a highly significant association with BMI-SDS.

**Conclusion:**

The present study demonstrated that siblings had a lower BMI-SDS than only children did. For single-born siblings, the association between birth order and increased BMI-SDS seemed to persist only up to 11 years of age.

## Introduction

Childhood obesity is among the most serious health challenges of this century [1]. In 2016, over 158 million children and adolescents aged 5–19 years were living with obese worldwide [2]. Moreover, nearly 90% of children with obesity by age 3 and more than half (60%) of pre-pubertal children with overweight will have overweight in early adulthood [3], demonstrating the importance of childhood for the development of obesity and the formation of habits to prevent it. Therefore, there is an urgent medical need to identify the precise risk factors for the development of obesity and to give recommendations to families at high risk.

Siblings living together in a family have a similar genetic background and grow up in the same social environment. Likewise, the family determines the food environment, including eating habits, and the level of physical activity until adolescence [4, 5]. Nevertheless, each family member has an individual health status, which is reflected, for example, in differences in BMI between siblings [6].

Yet, there is still a lack of knowledge on the underlying processes that lead to BMI differences between siblings, focussing on pre- and postnatal risk factors. In the pre- and perinatal period, maternal prepregnancy weight and weight gain during pregnancy are important, both of which may directly impact children’s birth weight [7]. Thereby, a higher birth weight is directly associated with a higher BMI later on, while a lower birth weight appears to be associated with increased central obesity [8].

In the postnatal phase, overweight and obesity depend on the interaction between the individual predisposition and environmental factors stemming from an imbalance between energy intake and energy consumption. Both individual and environmental factors are intertwined with the family’s health behaviour. For example, studies have shown that breastfed children are at lower risk for overweight and obesity than their never-breastfed peers [9], with this effect lasting into adolescence [10]. Further, more physical activity (PA) in everyday life promotes weight loss and decreased risk of overweight [11]. Here, the low or high PA of family members and peers can influence a child’s PA negatively as well as positively. Similarly, changes in family structure, such as an increase in the number of siblings and the timing of becoming a sibling, have been shown to affect later BMI [12, 13].

Most of the previous studies that analysed pre- or postnatal risk factors for childhood obesity focused on the role of socioeconomic status. Only a few studies have considered the association of siblings and birth order on weight status [14, 15], but even those do not consider the intra-family setting. In addition, results vary across different regions and time periods [16, 17]. Previous studies also failed to analyse BMI development over the entire period into adulthood for each location in the birth order. Instead, they usually focused on specific age groups and the total number of siblings [18, 19]. Thus, there is still a research gap regarding pre- and postnatal factors that might cause differences in BMI of siblings and whether there are birth order-specific dependencies.

Therefore, the present study compared the BMI in different sibling constellations by considering the child’s individual family membership. Our aim was to analyse weight differences between siblings, variations in BMI at different ages, and risk factors that might be associated with these differences. In addition, we differentiated well-known pre- and postnatal risk factors related to birth order, such as pregnancy-related factors, the age gap, breastfeeding, and children’s PA. Our research hypothesis postulated that having siblings significantly affect BMI-SDS, and first-born children have the lowest risk of developing overweight.

## Methods

### Study population

Data were obtained from the LIFE Child study, an ongoing longitudinal cohort study conducted in Leipzig, Germany, and the surrounding area [20, 21]. The LIFE Child study aims to monitor child development from birth into adulthood. Since 2011, participants have been recruited between the 24^th^ week of pregnancy and 16 years of age, with subsequent annual follow-ups.

Since participants were examined between one and eight times depending on their duration of study participation, the longitudinal design of this study is combined with cross-sectional analysis in certain cases. The overall average number of BMI measurements in the present study was 3.6 times; for only children, the average was 3.4.

In addition to the general exclusion criteria of LIFE Child, such as chronic, chromosomal or syndromal diseases, the present study excluded 95 children which were born prematurely or born to mothers diagnosed with gestational diabetes (irrespective of which pregnancy) or who had taken drugs during pregnancy (n = 18). Besides this, 101 children with missing values on gestational age and 5 triplet children were excluded. 212 half- and step-siblings (but not their families as a whole) were excluded because the sample sizes did not allow for correcting for different genetic relationships. The final study sample included n = 1932 children from 1194 families. Children were assigned to one of three subgroups: only children (children without siblings, n = 526), full siblings from single pregnancies (single-born siblings, n = 1348), and twin children (n = 58). Siblings were ranked as first-, second-, third-or-later-born and were additionally classified as having an age gap of ≤3 years or more than 3 years.

### Measurements

Values for body weight and height were collected at the LIFE Child Study Centre by trained staff during each study visit. Birth weight and length were obtained from the children’s medical records (well-child check-ups). The body length of children up to one year of age was determined using the stadiometer “Dr. Keller II” and body weight using the infant scale “seca 752”. Height and body weight of older children were assessed using the stadiometer “Dr. Keller I” and the scale “seca 701”, respectively. The body mass index (BMI) was calculated by dividing the weight by height squared and is given in kg/m^2^. Weight, height and BMI data were transformed into gender- and age-adjusted standard deviation scores (SDS) according to the German Working Group on Childhood and Adolescent Obesity (AGA) using German growth standards by Kromeyer-Hauschild [22, 23]. The overweight group consisted of children with a BMI-SDS ≥ 1.282 (90^th^ percentile) and included children with obesity: BMI-SDS ≥ 1.881 (>97^th^ percentile) [23].

The number of younger and older siblings, parents’ year of birth, socioeconomic status (SES), physical activity (PA) and breastfeeding were reported by the parents via online questionnaires. The Winkler Index, which is based on household income, parental education and occupational prestige, was used to classify SES as low, medium or high [24]. Maternal education level was stratified into two groups: at least a 10^th^ grade completion certificate (94% of all participating mothers, n = 1077), equivalent to a high school diploma for students not seeking to attend university, and 6% with a lower level of education (n = 70).

The duration of breastfeeding is based on the mother-reported values collected at each visit as long as the mother was breastfeeding. The last report given by the mother regarding duration of breastfeeding was used in the analysis. For children included in the study later, after ablactation, the duration of breastfeeding was collected at the first visit. Breastfeeding was recorded as the number of months of exclusive breastfeeding, and in addition, the total duration of breastfeeding.

Week of delivery, mother’s weight before pregnancy, mother’s weight during pregnancy and maternal height were taken from the mother’s medical records. Pregnancy weight gain was calculated as the difference between the weight recorded during the last visit before delivery and prior to pregnancy. Weight gain was included in the analysis only if the last measurement was obtained fewer than 21 days before the expected delivery date. If maternal prepregnancy weight was missing, the earliest weight measured before the end of week 12 of pregnancy was used as a substitute.

The age gap was defined as the age difference between a child and his or her next older sibling. For first-borns, the gap to the second-born was used.

Physical activity was assessed with three questions: 1. How often do you play outdoors? 2. How often do you participate in organised physical activity (e.g., a sports team)? 3. How often do you participate in non-organized physical activity? Five answer categories were available: “never”, “less than once a week”, “1 to 2 times a week”, “3 to 5 times a week”, and “almost every day”. For analysis, these were aggregated to 1) Never, 2) Up to 2 times/week and 3) 3–7 times/week.

### Statistical analysis

Descriptive statistics were reported as mean and standard error for continuous variables and counts and percentages for categorical variables.

The family was included as a random effect in all models to account for the dependency structure imposed by having multiple children per family. Moreover, to adjust for multiple measures per child, the child was also included as a random factor nested within the family. Therefore, it was possible to model the outcomes longitudinally. First, gestational age, birth length, birth weight, BMI-SDS, maternal and paternal age, maternal prepregnancy BMI, pregnancy weight gain and breastfeeding were each assessed depending on the birth order in separate univariate models. Second, the effects on BMI-SDS were each modelled in separate univariate analysis depending on the birth weight, maternal prepregnancy BMI, pregnancy weight gain, maternal age, breastfeeding, and physical activity. Furthermore, a multivariate analysis was carried out to analyse the joint effects on BMI-SDS by the factors birth order, birth weight, ever breastfed, child play outdoors and participation in organised PA. Calculations using mixed models can be stored in a special SAS item store. The effect plots were generated based on the content of the respective SAS item stores in a statistical post-fitting analysis. In the univariate models, birth order was set as the dependent variable and “first-born” as the reference category. For all other analysis, the category with the highest number of available cases was set as the reference.

For calculated percentages of birth weight group, low parental SES and low maternal education, statistical significance were analysed with a chi-squared test and Fisher’s Exact Test (twins). All other analyses were conducted and visualised using SAS Studio Version 3.8 (SAS Institute, Cary, NC, USA). A *p*-value < 0.05 was considered statistically significant. The analysis code is published at the following address: https://osf.io/kn8zc/.

### Ethical considerations

Written informed consent was obtained from all parents and all children older than age 12. The study and all procedures were in accordance with the ethical standards of the institutional and national research committee and with the Declaration of Helsinki (1964) and its later amendments. The study was approved by the local ethics committee (Reg. No. 264-10-19042010) and was registered in the National Clinical Trial Register (NCT02550236). Confidentiality was maintained during data analysis. The data were fully anonymized before interpretation. Applicable legal requirements and informed consent obtained allow public sharing of aggregated data only. For further information, LIFE Child (research@life-child.de) can be contacted directly.

## Results

The total analysed cohort consisted of n = 1932 children, 73% of whom were siblings and 27% only children. Birth year and gender distribution were comparable between the two groups (Table 1). The mean birth weight increased with birth order, with first-borns weighting ~180 g less than third-or-later-born children. Furthermore, first-borns had the smallest length at birth compared to second- and third-born children. BMI-SDS and percentage of children with overweight at ages 0–18 also showed a dependence on the birth order, with a strong positive trend from first-born to third-or-later-born children. First-born and only children had a comparable birth weight and height. However, only children showed the highest BMI-SDS and percentage of overweight at ages 0–18. Twins had the lowest birth weight, birth length and lowest BMI-SDS among all groups. Only children and third-or-later-born children had the highest percentages of low parental socio-economic status (SES) and low maternal education.

**Table 1:** Sample characteristics by sibling status.

Characteristics		Only child	First-born	Second-born	Third-or-later-born	Twins
Participants (n, %)		526 (27.2%)	578 (29.9%)	608 (31.5%)	162 (8.4%)	58 (3%)
Gestational age (weeks)		39.5±0.1* (n = 526)	39.7±0.0 (n = 578)	39.7±0.0 (n = 608)	39.5±0.1 (n = 162)	37.7±0.1*** (n = 58)
Gender male %		51.52	51.9	51.64	53.09	48.28
Birth year range		1994–2015	1994–2013	1994–2016	1995–2016	1998–2019
Birth length (cm) ± SEM		50.3±0.1 (n = 526)	50.4±0.1 (n = 567)	50.8±0.1*** (n = 604)	50.8±0.2** (n = 159)	47.5±0.3*** (n = 58)
Birth length-SDS		-0.44 (n = 524)	-0.40 (n = 557)	-0.22*** (n = 598)	-0.24** (n = 159)	-1.66*** (n = 58)
Birth weight (kg) ± SEM		3.43±0.02 (n = 525)	3.46±0.02 (n = 575)	3.59±0.02*** (n = 606)	3.64±0.04*** (n = 161)	2.69±0.04*** (n = 58)
Birth weight SDS		0.16 (n = 523)	0.23 (n = 565)	0.51*** (n = 600)	0.62*** (n = 160)	-1.36*** (n = 56)
Birth weight group (%) ^a^	SGA	5*** (n = 28)	6 (n = 32)	2*** (n = 10)	2 (n = 3)	47*** (n = 14)
	AGA	84*** (n = 439)	81 (n = 456)	78*** (n = 466)	72 (n = 115)	53*** (n = 16)
	LGA	11*** (n = 56)	14 (n = 78)	21*** (n = 124)	26 (n = 42)	0*** (n = 0)
Overweight or obese aged 0–18 years (%)^b^		24	12	14	19	9
BMI-SDS		0.13*** (n = 1811)	-0.05 (n = 2072)	0.03 (n = 2151)	0.15* (n = 616)	-0.18 (n = 205)
Low parental SES (%)		13*** (n = 62)	5 (n = 26)	5 (n = 32)	13*** (n = 21)	4 (n = 2)
Low maternal education (%)^c^		6 (n = 31)	5 (n = 26)	4 (n = 26)	13*** (n = 20)	0 (n = 0)

n = number of available cases; values are expressed as mean or mean ± SEM

^a^ LGA: birth weight-SDS ≥ 1.28, AGA: birth weight-SDS ≥ −1.28 to 1.28, SGA: birth weight-SDS < −1.28

^b^ BMI-SDS of 1.282 or more, a child who exceeded this value at least once is counted one time in the statistics

^c^ Percentage of mothers with less than a 10^th^ grade education. Significance in birth weight, low parental SES and low maternal education were analysed using a chi-squared test and Fisher’s exact test for twins. Means were significantly different from those in the reference group of first-borns

* p<0.05

** p<0.01

*** p<0.001. Abbreviations: SDS, standard deviation score; SEM, standard error of the mean; LGA, large for gestational age; AGA, appropriate for gestational age; SGA, small for gestational age.

### Siblings and birth order is associated with BMI

**Fig 1:**
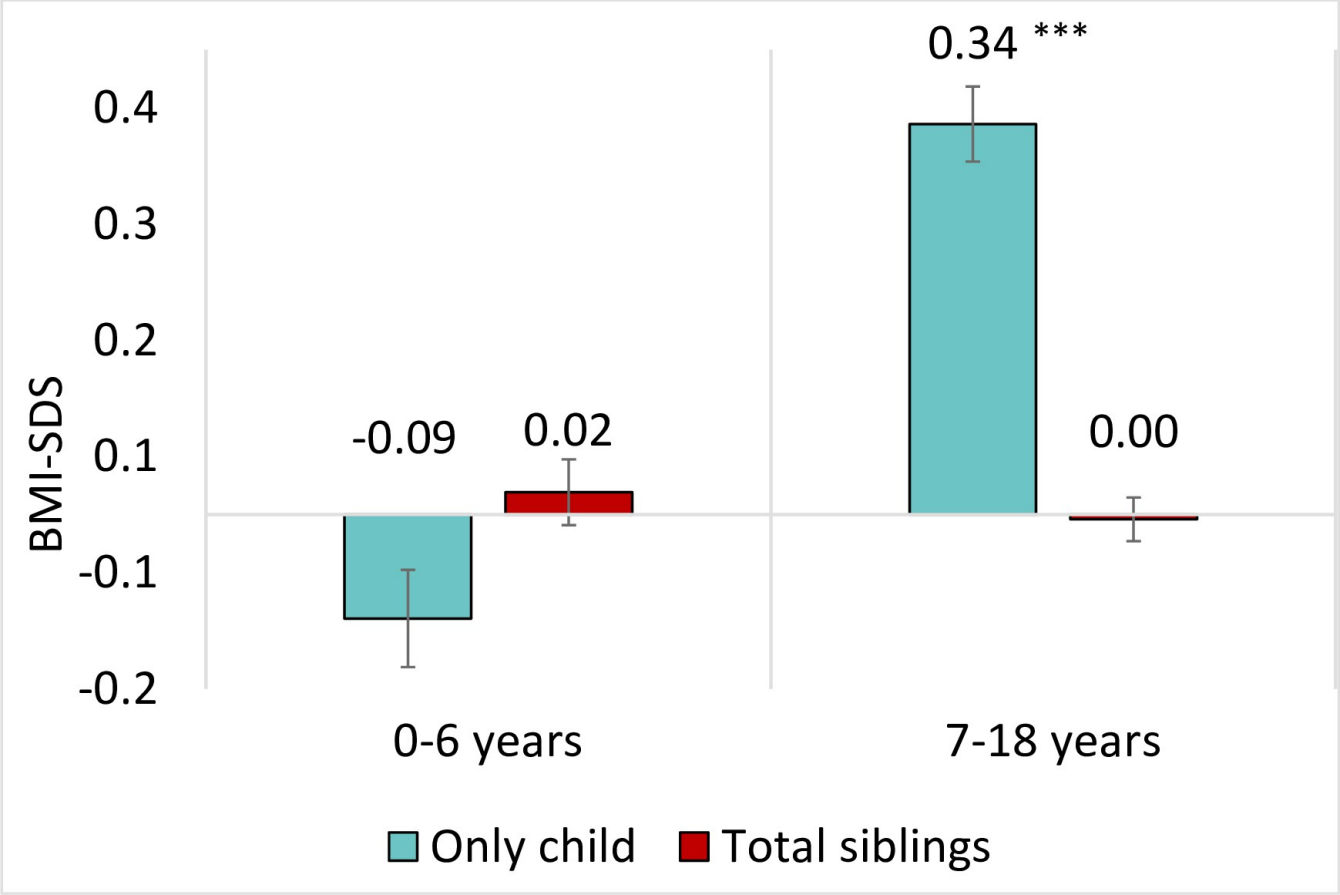
BMI-SDS by age range and sibling status. In each age range, the statistical reference group was “total siblings”. Significant differences between only children and children with siblings are presented as means with a vertical error indicator (standard error of the mean): ***p<0.001. Abbreviations: SDS, standard deviation score.

The group of “Total siblings” included first-born, second-born, third-or-later-born children and twins to assess if siblings have an effect on BMI-SDS (Fig 1). In early childhood, at age of 0–6 years, no significant difference in BMI-SDS between siblings and only children was observed. In contrast, at ages 7–18, the BMI-SDS was significantly higher in only children than in siblings.

**Fig 2:**
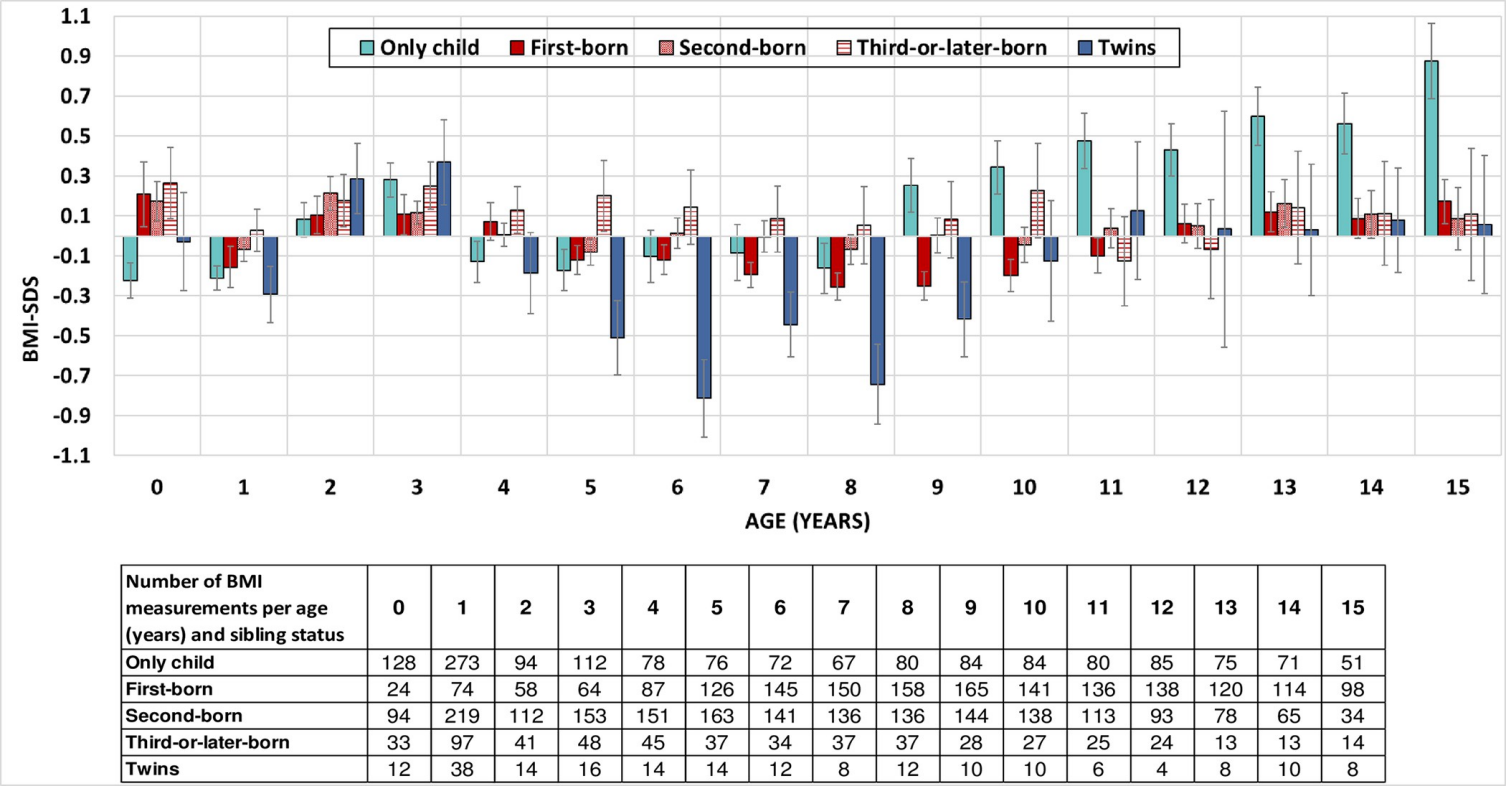
BMI-SDS by age and sibling status. BMI standard deviation scores at 1-yearintervals are shown according to sibling status: first-born, second-born, third-or-later-born, twins and only children. The values are means with a vertical error indicator (standard error of the mean). The number of measurements per age and sibling status is shown in the table.

**Fig 3:**
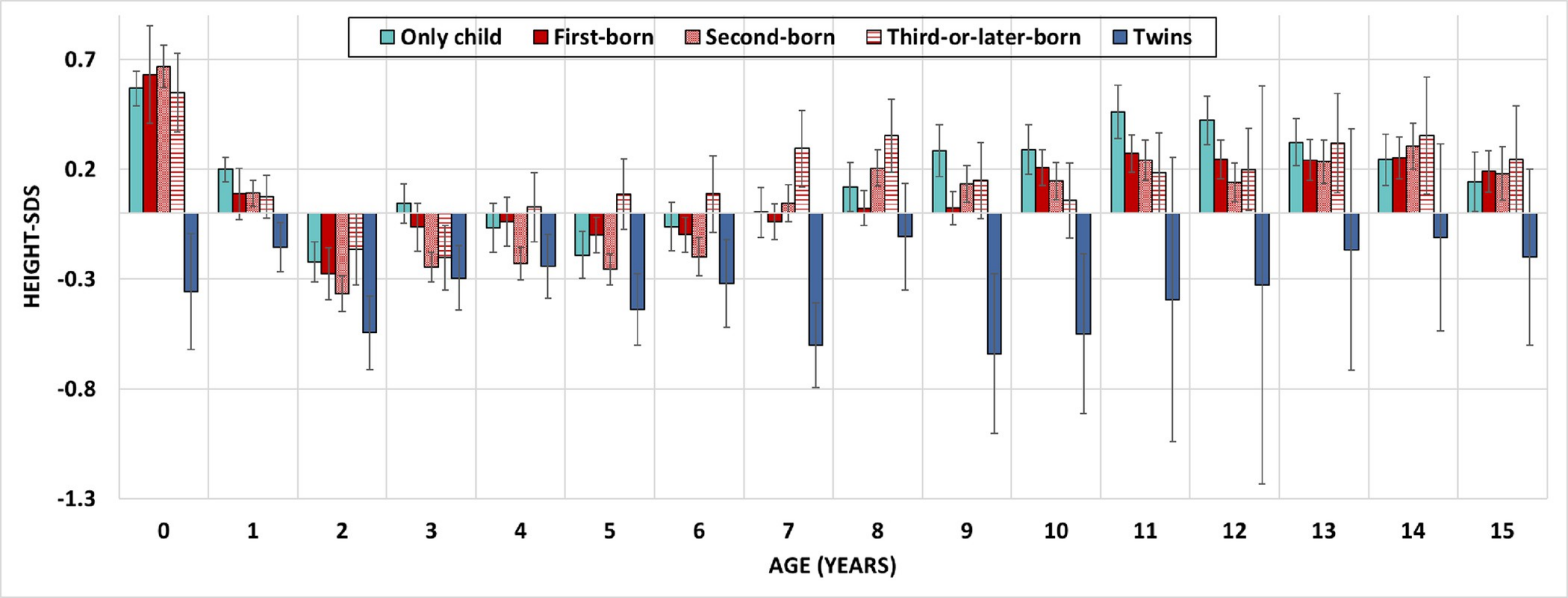
Height-SDS by age and sibling status. Height standard deviation scores in 1-yearintervals are shown according to sibling status: first-born, second-born, third-or-later-born, twins and only children. The values are means with a vertical error indicator (standard error of the mean).

We then specified the association between BMI-SDS and sibling status by evaluating annual measurements at 0–15 years of age and stratifying siblings according to their birth order (Fig 2). At 0–8 years of age, only children and first-borns had a comparable BMI-SDS. From 9 years of age on, the BMI-SDS of only children increased steadily, while this was not found in siblings. At around 15 years of age, the average BMI-SDS of only children was 0.9 SDS higher than the reference values. Up to ten years of age, third-or-later-born children showed the highest BMI-SDS, twins the lowest values. From eleven years of age onwards, the BMI-SDS of siblings and twins was comparable and was in a healthy range, about 0.1 SDS higher than the reference values. The increase in only children’s BMI could be mainly attributed to an increase in weight, as the height-SDS was comparable to first-borns (Fig 3).

### Effect of risk factors on BMI-SDS

**Fig 4:**
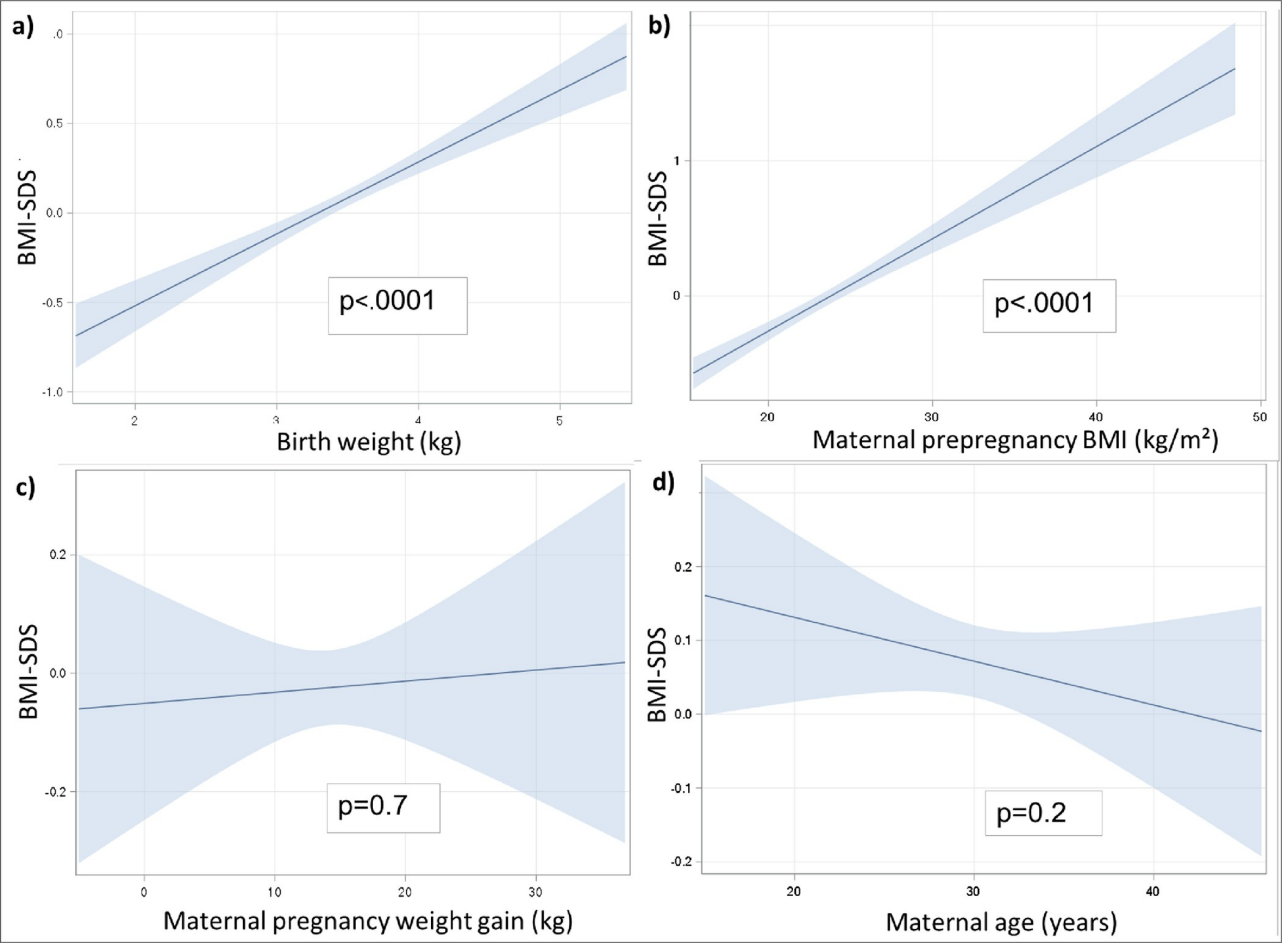
Effect plots of risk factors on BMI-SDS. Predicted values of BMI standard deviation score plotted against one continuous covariate/risk factor: a) birth weight; b) maternal prepregnancy BMI; c) maternal pregnancy weight gain; and d) maternal age. The calculations were made with mixed models, with the results stored in a special SAS item store; based on this calculation, the effect plots were generated in a statistical post-fitting analysis. 95% confidence intervals are shown as blue shade. Significance levels are shown in the plot for each respective risk factor. Significance was reached when: *** p<0.001.

Initially, we reviewed known factors for overweight for their risk potential among our study population. A high birth weight and high maternal prepregnancy BMI were significantly associated with the child’s BMI-SDS (Fig 4). Maternal pregnancy weight gain and maternal age at birth did not show significant associations with the child’s BMI-SDS in univariate analyses.

**Fig 5:**
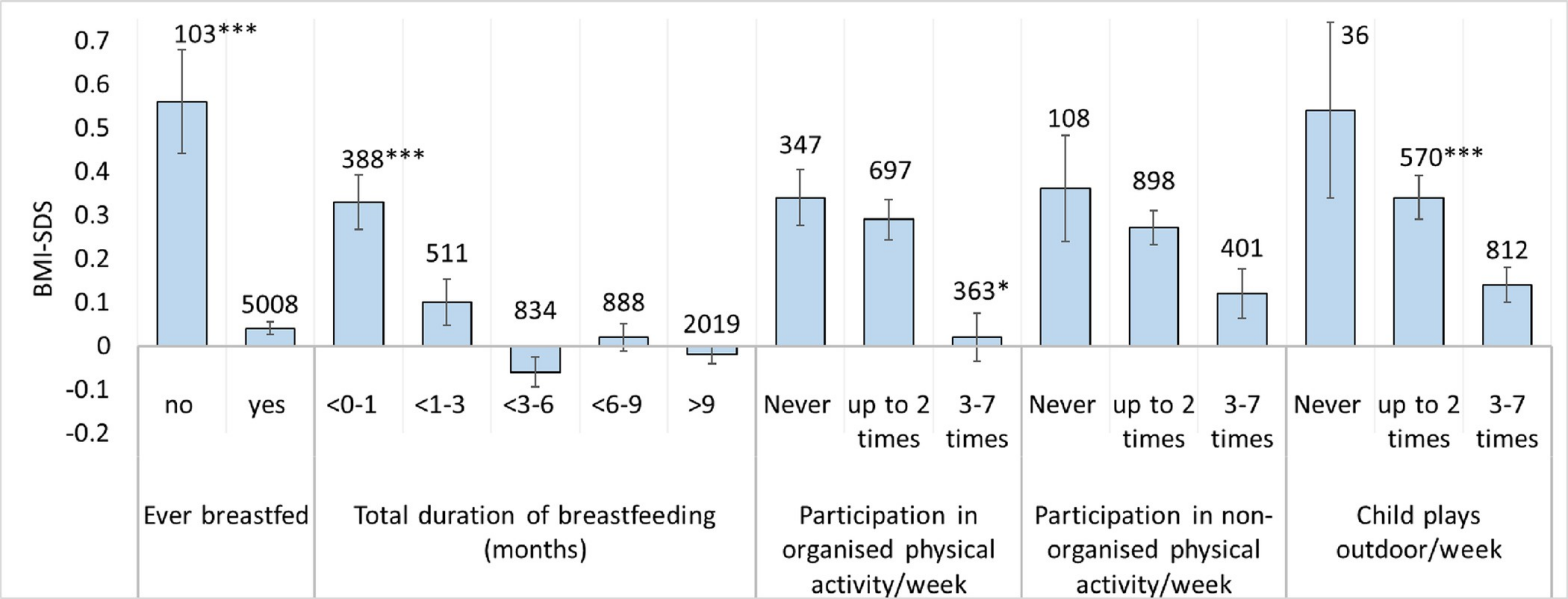
Relation between BMI-SDS and breastfeeding and physical activity. Associations between two categories of breastfeeding and three categories of physical activity with BMI standard deviation scores. The values are means with a vertical error indicator (standard error of the mean). The labelling of the bars represents the respective number of cases analysed. Statistically significant difference in means was considered as *p<0.05, ***p<0.001 using mixed models. The statistical reference category was the group with the highest number of answers: “Up to 2 times/week” for the categories “Participation in organised physical activity” and “Participation in non-organised physical activity”; “3–7 times/week” for the category “Playing outdoors”.

In the postnatal state, BMI-SDS can be affected by the duration of breastfeeding and by physical activity. BMI-SDS was 0.5 SDS higher in children who were never breastfed. Particularly in the first months of life, breastfeeding appeared to be associated with a lower BMI-SDS, with a significant difference (p<0.001) from 0–1 months to > 9 months (Fig 5). Each of the three PA categories showed a trend in which increased time spent at play or engaged in sports was associated with lower BMI-SDS.

### Risk factors of overweight differentiated by birth order

#### Prenatal risk factors

Mothers of first-born children had the lowest prepregnancy BMI (Table 2). As previously shown [25], maternal pregnancy weight gain was significantly lower (p<0.001) with increasing birth order. The prepregnancy BMI of mothers of only children, second- and third-or-later-born children was comparable. Twins showed the highest maternal prepregnancy BMI and maternal weight gain.

**Table 2:** Parental characteristics by sibling status.

Characteristics	Only child	First-born	Second-born	Third-or-later-born	Twins
Maternal age (years)	29.3±0.2*** (n = 450)	28.0±0.2 (n = 547)	31.3±0.2*** (n = 576)	33.9±0.3*** (n = 158)	32.0±0.7*** (n = 58)
Paternal age (years)	32.6±0.3*** (n = 436)	30.4±0.2 (n = 547)	33.9±0.2*** (n = 576)	37.1±0.5*** (n = 158)	33.9±0.7*** (n = 56)
Maternal prepreg-nancy BMI (kg/m^2^)	23.3±0.3 (n = 268)	22.7±0.2 (n = 286)	23.5±0.2*** (n = 355)	23.3±0.5*** (n = 68)	25.4±1.3 (n = 27)
Maternal pregnan-cy weight gain (kg)	15.0±0.3 (n = 221)	14.7±0.3 (n = 272)	13.3±0.3*** (n = 284)	12.9±0.5*** (n = 63)	16.4±1.5 (n = 8)

The table contains the parental characteristics stratified by sibling status. Values are means *±* standard error of the mean. The numbers in parentheses correspond to the respective available number of cases. Significance was tested univariately with a mixed model, with first-born children as the reference group. Significant differences were marked with *** p<0.001.

#### Age gap between siblings

To test whether a larger age gap between siblings is associated with BMI-SDS, we divided the siblings into two groups: children in the first group had an age gap of ≤3 years (n = 795 children), and those in the second group had an age gap of >3 years (n = 521 children) to their next-oldest sibling. The average age gap was about 4 years. An age gap >3 years increased the BMI-SDS for second- and third-or-later-born children, without reaching significance (Table 3). The BMI-SDS for first-borns remained almost the same regardless of age gap. However, independent of the age gap, the overall dependence of BMI-SDS on birth order was still present. A bivariate statistical model with birth order as covariate did not reveal a significant relationship between age gap and BMI-SDS (*p* = 0.29).

**Table 3:** Relationship between BMI-SDS and age gap of siblings.

	Age gap ≤3 years	Age gap >3 years
**First-born**	-0.03±0.03 (n = 926)	-0.04±0.04 (n = 535)
**Second-born**	0.03±0.03 (n = 900)	0.06±0.04 (n = 643)
**Third-or-later-born**	0.06±0.06 (n = 245)	0.24±0.08 (n = 189)

The table compares two groups, stratified according to birth order, in which siblings have an age difference of less or more than 3 years. Values are means ± standard error of the mean. The numbers in parentheses corresponds to the respective number of cases. First-born children served as the statistical reference group for each age gap group. SDS, standard deviation score.

#### Postnatal risk factors

Single-born siblings had the highest rates and longest time periods of breastfeeding (Table 4). Exclusive and total duration of breastfeeding were significantly lower for only children and twins compared to single-born siblings. Total duration of breastfeeding was significantly associated with birth order. Third-or-later-born children were breastfed two months longer than other birth order groups.

**Table 4:** Duration of breastfeeding by sibling status.

Process parameters	Only child	First-born	Second-born	Third-or-later-born	Twins
Ever breastfed (%)	95.1 (n = 526)	95.8 (n = 577)	97.4 (n = 606)	96.3 (n = 162)	91.7 (n = 48)
Exclusive breastfeeding (months)	3.8*** (n = 501)	4.6 (n = 572)	4.6 (n = 583)	4.8 (n = 156)	2.4*** (n = 38)
Total duration of breastfeeding (months)	7.2*** (n = 489)	8.8 (n = 553)	8.9 (n = 575)	10.6* (n = 153)	5.8* (n = 42)

Values are means ± standard error of the mean. The numbers in parentheses correspond to the respective available number of cases. First-born children served as the statistical reference group, significant difference of means was considered

*p<0.05

**p<0.01

***p<0.001. SDS, standard deviation score.

**Fig 6:**
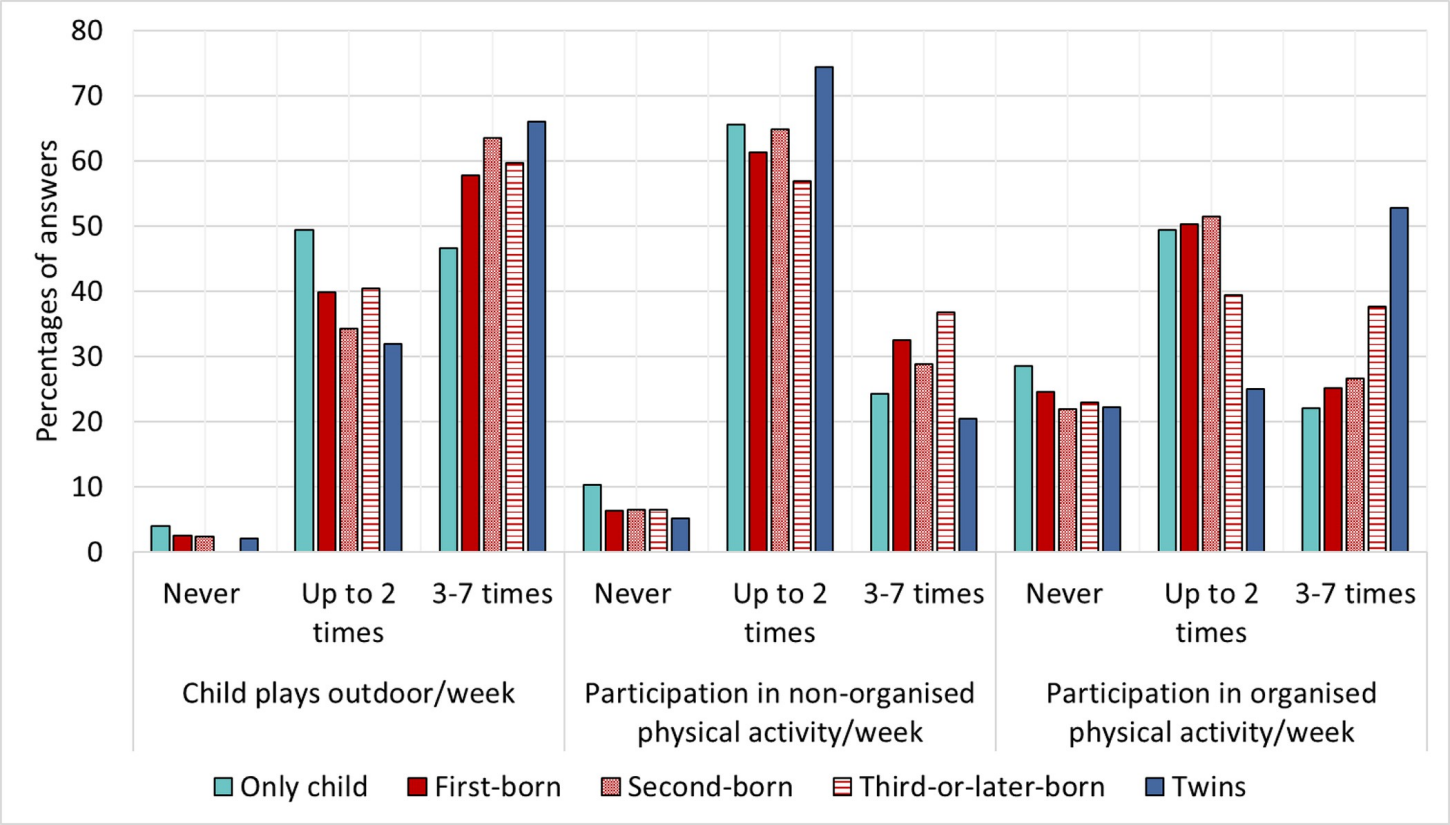
Assessment of physical activity in three categories by sibling status. The graph shows the percentage of children per sibling status selecting the three activity categories “Child plays outdoor”, “Participation in non-organised physical activity” and “Participation in organised physical activity”. The answer categories were aggregated into three groups: 1) Never, 2) Up to 2 times/week and 3) 3–7 times/week.

Compared to the sibling groups, only children had the lowest PA, with the highest percentage selecting "never" and the lowest percentage selecting “3–7 times/week” in all three activity categories. Twins and third-or-later-born children had the highest percentages of “3–7 times/week” in all three categories. For single-born siblings, PA seemed to be dependent on birth order: first-born children were less active, followed by second-born. The most active group were third-or-later-born children (Fig 6).

### Simultaneous analysis of significant risk factors on BMI

We next used multivariate analysis to adjust for confounding factors on BMI-SDS (Table 5). Birth weight, ever being breastfed and playing outdoors showed highly significant associations with BMI-SDS. The largest difference was found between only children and first-borns, with beta = 0.6, p<0.001.

**Table 5:** Multivariate analysis of the effect size order of postnatal risk factors on BMI-SDS.

Confounder		Estimate (Standardised Beta Coefficient)	*p*-Value
**Birth order**	**Only child**	0.64	<0.001***
	**First-born**	0	Reference
	**Second-born**	-0.01	0.9
	**Third-or-later-born**	-0.01	0.9
	**Twins**	0.36	0.3
**Birth weight**		0.20	<0.001***
**Ever breastfed**		-0.11	<0.001***
**Child plays outdoors**		-0.03	0.002**
**Participation in organised physical activity**		-0.02	0.1

Means were significant at

** p<0.01

*** p<0.001.

## Discussion

The present study showed for the first time that in an intra-family setting, the BMI of siblings at different locations within the birth order becomes comparable in later childhood and lies within the healthy range. Although parameters at birth did not differ between only children and first-borns, BMI-SDS was significantly higher in only children in later childhood.

In early childhood up to age 11, children with siblings had lower BMI, particularly first- and second-born children. Of all siblings, third-or-later-born children had the highest BMI-SDS, which is in line with several previously published studies [18, 26]. These results confirmed our proposed research hypothesis, but only up to 11 years of age and not for the entire period up to adulthood. With an age gap of more than three years between siblings, higher BMI-SDS was shown in second-born and third-or-later-born children. One possible explanation is that children with a larger gap between births might grow up under slightly different family conditions, which may have an association with BMI at later stages in life [27]. Twins, with the smallest age gap, had only a slight difference in birth weight and BMI compared to single-born siblings [28].

The negative association between BMI and lacking siblings could be clearly observed in only children, who tended to gain much more weight, particularly in later childhood. Our results are consistent with previous studies demonstrating an increased risk of overweight in only children [29].

So far, there are only a few studies stating a specific time point at which only children start to have an increased risk of obesity. We showed for the first time that the BMI of only children increased significantly and steadily beginning at 9 years of age, and remained higher than that of siblings from that time onwards. Ikeda et al. [30] support the present findings by demonstrating that the excess risk of obesity among children living with no siblings increased between kindergarten and Grade 8. In a multivariate model, we were able to clarify that being an only child was an independent risk factor for an increased BMI-SDS, independent of the pre- or postnatal factors breastfeeding and physical activity.

By nature, prenatal conditions are similar for first-born and only children. In our study, the lowest maternal prepregnancy BMI and lowest birth weights were recorded for first-born and only children. Both parameters were also confirmed as risk factors for overweight, which is in line with previous studies [31, 32]. Further studies have shown that maternal pregnancy weight gain is positively associated with the infant’s birth weight and BMI-SDS [33, 34]. However, we have previously shown that with birth order, birth weight increases and the mother gains less weight during pregnancy [25]. Further, a univariate analysis showed no significant association between maternal pregnancy weight gain and BMI in general. Badon et al. partially support our findings by comparing siblings and showing that maternal gestational weight gain was associated with a higher birth weight, but not with the child’s BMI [35].

Most likely, factors other than prenatal factors and birth weight could have contributed to the BMI differences between siblings and only children. Postnatal risk factors seem to have a stronger association with BMI-SDS difference between first-borns and only children in adolescence. We therefore investigated two known postnatal factors individually by birth order to clarify their influence on BMI: breastfeeding and physical activity. The duration of breastfeeding was significantly lower for only children compared to single-born siblings. Exclusive breastfeeding and duration of breastfeeding were also positively associated with birth order. Breastfed children were generally about 0.52 SDS thinner than non-breastfed children, supporting previous findings [36]. How specific alkylglycerols (AKG), which are only present in breast milk, prevent the formation of lipid-storing white adipose tissue has already been described previously [37]. A longer breastfeeding duration therefore also enables the intake of these AKGs over a longer period of time, which reduces overweight [38] and could lead to a lower risk of having overweight as an adult [39]. Thus, the approach of BMI-SDS of third-or-later-born children was likely achieved because they had the longest breastfeeding period, of almost eleven months, and the highest physical activity.

Studies have shown that children with siblings generally have higher activity levels [40], particularly children with older siblings [41]. Siblings can be playmates and encourage active participation in sports. Siblings also act as additional caregivers, which sometimes makes active play possible in the first place. It is therefore not surprising that having fewer siblings is associated with reduced physical activity [6]. The results of our study confirm these findings, as only children had the lowest activity. A general decrease in activity levels among children from age 7 onwards [42] may have additionally contributed to the steady increase in BMI among only children. Twins, along with third-born children, tended to participate in physical activity very often. As twins start with a low birth weight, one might assume that their later BMI is also low. Nevertheless, the BMI of twins approached that of single-born children with siblings. The negative association between a short breastfeeding period and BMI could also contribute here, while the positive association with having a sibling should also be considered.

However, some studies also relate physical activity to socioeconomic status (SES). In the present study, the highest percentage of low parental SES was found among only children and third-or-later-born children, with only children the least physically active and third-or-later-born children the most active group. In contrast, a Swedish study showed that children from low-SES families were more physically active, but also twice as likely to have overweight as children from high-SES families [43]. Low parental education, an indicator of SES, is known to have a significant positive association with BMI-SDS, with the effect strongest for children with the highest BMI-SDS [44]. These data are in line with the present study’s findings. However, the findings of the Swedish study were not confirmed here for third-born children, even though a high percentage of them grew up in families with low SES, as third-or-later-born children developed a healthy BMI in later childhood. Thus, including the family as a random effect led to more differentiated results in the present study. We thus conclude that controlling for sibling distribution is advisable in further studies.

Among the strengths of this large sample size study was the reduction of potential heritable and socio-cultural confounders. However, this required the exclusion of half- and step-siblings, which depended on the accuracy of parents’ reports. Including children ranging in age from 0–18 years enables a differentiated assessment of weight development over the entire infancy, childhood and adolescent periods. Further, examining only children in a separate group made it possible to compare them with first-borns with regard to both pre- and postnatal risk factors.

The present study also has some limitations: Like other observational studies, the results may be biased by factors that were not directly observed. For example, we did not control for maternal smoking during pregnancies. Children whose mothers smoked have a lower birth weight SDS, and through a catch-up process, eventually have a higher BMI-SDS compared to the children of non-smoking mothers [45]. However, we excluded children who were born prematurely, which often occurs in mothers who smoke. Postnatally, children’s eating habits after breastfeeding were not studied. For example, maternal snack offers might differ according to birth order [46]. Furthermore, the influence of half- and step-siblings on pre- and postnatal risk factors were not analysed. We might speculate that older half-siblings with the same mother may change the prenatal influences or breastfeeding duration due to the birth order. Likewise, other children in the household, older or younger, can influence the physical activity and eating habits of all children. Further, the need for voluntary and active participation in the present cohort studies shifted the cohort towards a higher socio-economic status. This is underlined by the fact that 93% of all participating mothers had at least a 10th grade completion certificate. However, third- or later-born children had the significantly highest percentages of low parental socioeconomic status (SES) and lower maternal educational attainment, which may contribute to the increased BMI of this group.

In conclusion, the results of the present study demonstrated that children’s BMI strongly depended on sibling status. We found support for our proposed hypothesis that siblings have a significant impact on BMI-SDS, and first-born children had the lowest risk up to 11 years of age. At older ages, children with siblings had a lower BMI than only children, and BMIS-SDS only slightly differed by birth order. Postnatally, the longer breastfeeding duration and higher physical activity of single-born siblings may have contributed to their healthier BMI compared to only children. For only children, postnatal factors were associated with a strong increase in BMI. The data from the present study could be useful for preventing or treating obesity in families at high risk. Growing up as an only child could be considered a novel risk factor for an increased BMI and later obesity.

## References

[1] Nittari G, Scuri S, Petrelli F, Pirillo I, di Luca NM, Grappasonni I. Fighting obesity in children from European World Health Organization member states. Epidemiological data, medical-social aspects, and prevention programs. Clin Ter. 2019;170(3):e223–e30. doi: 10.7417/CT.2019.2137 31173054

[2] Lobstein T., H. B. Atlas of Childhood Obesity. www.worldobesity.org: World Obesity Federation; 2019.

[3] Geserick M, Vogel M, Gausche R, Lipek T, Spielau U, Keller E, et al. Acceleration of BMI in Early Childhood and Risk of Sustained Obesity. N Engl J Med. 2018;379(14):1303–12. doi: 10.1056/NEJMoa1803527 30281992

[4] Chen AY, Escarce JJ. Family structure and childhood obesity, Early Childhood Longitudinal Study—Kindergarten Cohort. Prev Chronic Dis. 2010;7(3):A50. 20394689 PMC2879982

[5] Gruber K, Haldeman L. Using the Family to Combat Childhood and Adult Obesity. Prev Chronic Dis. 2009;6(3). 19527578 PMC2722397

[6] Park SH, Cormier E. Influence of Siblings on Child Health Behaviors and Obesity: A Systematic Review. Journal of Child and Family Studies. 2018.

[7] Simmons R. Perinatal programming of obesity. Seminars in perinatology. 2008;32(5):371–4. doi: 10.1053/j.semperi.2008.08.004 18929161 PMC3357631

[8] Oken E, Gillman MW. Fetal origins of obesity. Obes Res. 2003;11(4):496–506. doi: 10.1038/oby.2003.69 12690076

[9] Koletzko B, Godfrey KM, Poston L, Szajewska H, van Goudoever JB, de Waard M, et al. Nutrition During Pregnancy, Lactation and Early Childhood and its Implications for Maternal and Long-Term Child Health: The Early Nutrition Project Recommendations. Ann Nutr Metab. 2019;74(2):93–106. doi: 10.1159/000496471 30673669 PMC6397768

[10] Hunsberger M, Consortium I. Early feeding practices and family structure: associations with overweight in children. Proc Nutr Soc. 2014;73(1):132–6. doi: 10.1017/S0029665113003741 24507855

[11] Wirth A, Wabitsch M, Hauner H. The prevention and treatment of obesity. Dtsch Arztebl Int. 2014;111(42):705–13. doi: 10.3238/arztebl.2014.0705 25385482 PMC4233761

[12] Dasgupta K, Solomon KT. Family size effects on childhood obesity: Evidence on the quantity-quality trade-off using the NLSY. Econ Hum Biol. 2018;29:42–55. doi: 10.1016/j.ehb.2018.01.004 29428886

[13] Wang H, Sekine M, Chen X, Kanayama H, Yamagami T, Kagamimori S. Sib-size, birth order and risk of overweight in junior high school students in Japan: results of the Toyama Birth Cohort Study. Prev Med. 2007;44(1):45–51. doi: 10.1016/j.ypmed.2006.07.015 16952392

[14] Ochiai H, Shirasawa T, Ohtsu T, Nishimura R, Morimoto A, Obuchi R, et al. Number of siblings, birth order, and childhood overweight: a population-based cross-sectional study in Japan. BMC public health. 2012;12:766. doi: 10.1186/1471-2458-12-766 22966779 PMC3509397

[15] Mosli RH, Miller AL, Peterson KE, Kaciroti N, Rosenblum K, Baylin A, et al. Birth order and sibship composition as predictors of overweight or obesity among low-income 4- to 8-year-old children. Pediatric obesity. 2016;11(1):40–6. doi: 10.1111/ijpo.12018 25735955 PMC4558390

[16] Hunsberger M, Formisano A, Reisch LA, Bammann K, Moreno L, De Henauw S, et al. Overweight in singletons compared to children with siblings: the IDEFICS study. Nutr Diabetes. 2012;2:e35. doi: 10.1038/nutd.2012.8 23448718 PMC3408642

[17] Stettler N, Tershakovec AM, Zemel BS, Leonard MB, Boston RC, Katz SH, et al. Early risk factors for increased adiposity: a cohort study of African American subjects followed from birth to young adulthood. Am J Clin Nutr. 2000;72(2):378–83. doi: 10.1093/ajcn/72.2.378 10919930

[18] Hu J, Ding N, Zhen S, Liu Y, Wen D. Who is more likely to be obese or overweight among siblings? A nationally representative study in rural China. PloS one. 2017;12(11):e0187693. doi: 10.1371/journal.pone.0187693 29176827 PMC5703493

[19] Meller FO, Loret de Mola C, Assuncao MCF, Schafer AA, Dahly DL, Barros FC. Birth order and number of siblings and their association with overweight and obesity: a systematic review and meta-analysis. Nutr Rev. 2018;76(2):117–24. doi: 10.1093/nutrit/nux060 29315408

[20] Quante M, Hesse M, Dohnert M, Fuchs M, Hirsch C, Sergeyev E, et al. The LIFE child study: a life course approach to disease and health. BMC public health. 2012;12:1021. doi: 10.1186/1471-2458-12-1021 23181778 PMC3533937

[21] Poulain T, Baber R, Vogel M, Pietzner D, Kirsten T, Jurkutat A, et al. The LIFE Child study: a population-based perinatal and pediatric cohort in Germany. Eur J Epidemiol. 2017;32(2):145–58. doi: 10.1007/s10654-016-0216-9 28144813

[22] Kromeyer-Hauschild K, Wabitsch M, Kunze D, Geller F, Geiß HC, Hesse V, et al. Perzentile für den Body-mass-Index. Monatsschrift Kinderheilkunde. 2001:807.

[23] Wabitsch M, Moss A. Evidence-based (S3) guideline of the Working Group on Childhood and Adolescent Obesity (AGA) of the German Obesity Society (DAG) and the German Society of Pediatrics and Adolescent Medicine (DGKJ) https://www.awmf.org/leitlinien/detail/ll/050-002.html2019

[24] Lampert T, Kroll L, Muters S, Stolzenberg H. [Measurement of socioeconomic status in the German Health Interview and Examination Survey for Adults (DEGS1)]. Bundesgesundheitsblatt, Gesundheitsforschung, Gesundheitsschutz. 2013;56(5–6):631–6. doi: 10.1007/s00103-012-1663-4 23703479

[25] Bohn C, Vogel M, Poulain T, Spielau U, Hilbert C, Kiess W, et al. Birth weight increases with birth order despite decreasing maternal pregnancy weight gain. Acta Paediatr. 2021;110(4):1218–24. doi: 10.1111/apa.15598 32981144

[26] Haugaard LK, Ajslev TA, Zimmermann E, Angquist L, Sorensen TI. Being an only or last-born child increases later risk of obesity. PloS one. 2013;8(2):e56357. doi: 10.1371/journal.pone.0056357 23437116 PMC3577826

[27] Rokholm B, Silventoinen K, Tynelius P, Sorensen TI, Rasmussen F. Modifiable environmental influences on body mass index shared by young adult brothers. Int J Obes (Lond). 2013;37(2):211–5. doi: 10.1038/ijo.2012.151 22945609

[28] The NS, Adair LS, Gordon-Larsen P. A study of the birth weight-obesity relation using a longitudinal cohort and sibling and twin pairs. Am J Epidemiol. 2010;172(5):549–57. doi: 10.1093/aje/kwq169 20688900 PMC3025637

[29] Li M, Xue H, Wang W, Wen M, Wang Y. Increased obesity risks for being an only child in China: findings from a nationally representative study of 19,487 children. Public Health. 2017;153:44–51. doi: 10.1016/j.puhe.2017.07.002 28843799

[30] Ikeda N, Fuse K, Nishi N. Changes in the effects of living with no siblings or living with grandparents on overweight and obesity in children: Results from a national cohort study in Japan. PloS one. 2017;12(4):e0175726. doi: 10.1371/journal.pone.0175726 28414810 PMC5393582

[31] Yu Z, Han S, Zhu J, Sun X, Ji C. Pre-Pregnancy Body Mass Index in Relation to Infant Birth Weight and Offspring Overweight/Obesity: A Systematic Review and Meta-Analysis. PloS one. 2013;8(4):e61627. doi: 10.1371/journal.pone.0061627 23613888 PMC3628788

[32] Wang X, Zhang X, Zhou M, Juan J, Wang X. Association of prepregnancy body mass index, rate of gestational weight gain with pregnancy outcomes in Chinese urban women. Nutr Metab (Lond). 2019;16:54. doi: 10.1186/s12986-019-0386-z 31452666 PMC6700840

[33] Su WJ, Chen YL, Huang PY, Shi XL, Yan FF, Chen Z, et al. Effects of Prepregnancy Body Mass Index, Weight Gain, and Gestational Diabetes Mellitus on Pregnancy Outcomes: A Population-Based Study in Xiamen, China, 2011–2018. Ann Nutr Metab. 2019;75(1):31–8. doi: 10.1159/000501710 31302647

[34] Voerman E, Santos S, Patro Golab B, Amiano P, Ballester F, Barros H, et al. Maternal body mass index, gestational weight gain, and the risk of overweight and obesity across childhood: An individual participant data meta-analysis. PLoS Med. 2019;16(2):e1002744. doi: 10.1371/journal.pmed.1002744 30742624 PMC6370184

[35] Badon SE, Quesenberry CP, Xu F, Avalos LA, Hedderson MM. Gestational weight gain, birthweight and early-childhood obesity: between- and within-family comparisons. Int J Epidemiol. 2020. 10.1093/ije/dyaa110 PMC7746402 32830276

[36] Metzger MW, McDade TW. Breastfeeding as obesity prevention in the United States: a sibling difference model. Am J Hum Biol. 2010;22(3):291–6. doi: 10.1002/ajhb.20982 19693959

[37] Yu H, Dilbaz S, Cossmann J, Hoang AC, Diedrich V, Herwig A, et al. Breast milk alkylglycerols sustain beige adipocytes through adipose tissue macrophages. J Clin Invest. 2019;130:2485–99. doi: 10.1172/JCI125646 31081799 PMC6546455

[38] von Kries R, Koletzko B, Sauerwald T, von Mutius E, Barnert D, Grunert V, et al. Breast feeding and obesity: cross sectional study. BMJ. 1999;319:147–50. doi: 10.1136/bmj.319.7203.147 10406746 PMC28161

[39] Martorell R, Stein AD, Schroeder DG. Early nutrition and later adiposity. J Nutr. 2001;131(3):874S–80S. doi: 10.1093/jn/131.3.874S 11238778

[40] Kracht CL, Sisson SB. Sibling influence on children’s objectively measured physical activity: a meta-analysis and systematic review. BMJ Open Sport Exerc Med. 2018;4(1):e000405. doi: 10.1136/bmjsem-2018-000405 30364499 PMC6196974

[41] van Sluijs EM, McMinn AM, Inskip HM, Ekelund U, Godfrey KM, Harvey NC, et al. Correlates of light and moderate-to-vigorous objectively measured physical activity in four-year-old children. PloS one. 2013;8(9):e74934. doi: 10.1371/journal.pone.0074934 24040365 PMC3764204

[42] Farooq MA, Parkinson KN, Adamson AJ, Pearce MS, Reilly JK, Hughes AR, et al. Timing of the decline in physical activity in childhood and adolescence: Gateshead Millennium Cohort Study. Br J Sports Med. 2018;52(15):1002–6. doi: 10.1136/bjsports-2016-096933 28288966 PMC6204977

[43] Beckvid Henriksson G, Franzen S, Elinder LS, Nyberg G. Low socio-economic status associated with unhealthy weight in six-year-old Swedish children despite higher levels of physical activity. Acta Paediatr. 2016;105(10):1204–10. doi: 10.1111/apa.13412 27008097

[44] Beyerlein A, Toschke AM, von Kries R. Risk factors for childhood overweight: shift of the mean body mass index and shift of the upper percentiles: results from a cross-sectional study. Int J Obes (Lond). 2010;34(4):642–8. doi: 10.1038/ijo.2009.301 20084072

[45] Beyerlein A, Rückinger S, Toschke AM, Schaffrath Rosario A, Von Kries R. Is low birth weight in the causal pathway of the association between maternal smoking in pregnancy and higher BMI in the offspring? Eur J Epidemiol. 2011;26:413–20. doi: 10.1007/s10654-011-9560-y 21360298

[46] Damen FWM, Steenbekkers B, Fogliano V, Luning PA. Youngest versus oldest child: why does mothers’ snack choice differ? Appetite. 2020;144:104455. doi: 10.1016/j.appet.2019.104455 31521767

